# The journey to understanding incretin systems: Theory, practice and more theory

**DOI:** 10.1111/jdi.13123

**Published:** 2019-08-20

**Authors:** Daisuke Yabe, Hitoshi Kuwata, Yutaka Seino

**Affiliations:** ^1^ Department of Diabetes and Endocrinology Gifu University Graduate School of Medicine Gifu Japan; ^2^ Yutaka Seino Distinguished Center for Diabetes Research Kansai Electric Power Medical Research Institute Kobe Japan; ^3^ Division of Molecular and Metabolic Medicine Department of Physiology and Cell Biology Kobe University Graduate School of Medicine Kobe Japan; ^4^ Center for Diabetes, Metabolism and Endocrinology Kansai Electric Power Medical Research Institute Osaka Japan; ^5^ Center for Clinical Nutrition and Metabolism Kansai Electric Power Hospital Osaka Japan

## Abstract

Accumulating clinical data on incretin‐based dipeptidyl peptidase‐4 inhibitors and glucagon‐like peptide‐1 receptor agonists in the past decade have clearly confirmed their safety and efficacy as antidiabetes drugs. However, the journey to understand the incretin system and its role in health and disease continues.

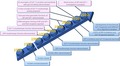

Type 2 diabetes in Asian patients is characterized by lack of obesity and impaired β‐cell function^1^, ^2^. Dipeptidyl peptidase‐4 (DPP‐4) inhibitors, which suppress degradation of the incretins glucose‐dependent insulinotropic polypeptide (GIP) and glucagon‐like peptide‐1 (GLP‐1) to potentiate insulin secretion glucose‐dependently, are therefore in wide use today in the management of type 2 diabetes in Asia^1^, ^2^.

Approximately 100 years have passed since the discovery of the incretin concept to clinical application as therapy in the treatment of diabetes (Figure 1)^3^. Inspired by Bayliss and Starling's discovery of secretin in 1902, Moore *et al*.^4^ hypothesized in 1906 that gut extracts contain a hormone that regulates the endocrine pancreas, and showed that administration of these gut extracts reduces the amount of urine sugars in patients with diabetes, presumably through stimulation of the endocrine pancreas. In 1929, La Barre purified the glucose‐lowering element from gut extracts, and named it incretin (INtestine seCRETtion Insulin). However, the hormone was forgotten for three decades until radioimmunoassay to measure insulin became available in the 1960s. Oral glucose load was then shown to produce a much greater insulin response than intravenous injection of glucose, which now can be attributed to action of the incretins. In the 1970s, GIP became the first identified incretin; intravenous administration of GIP was found to stimulate insulin secretion in healthy men, and was shown to act directly on isolated pancreatic islets to stimulate insulin secretion. As immunological depletion of GIP did not abolish all insulin‐stimulating activity of gut extracts, the existence of another incretin was inferred. Indeed, GLP‐1 secreted from L cells of the lower intestine and colon was found to act directly on islets to stimulate insulin secretion both in isolated pancreatic islets and healthy men. In the 1980s, GLP‐1 was thus identified as the second incretin.

**Figure 1:**
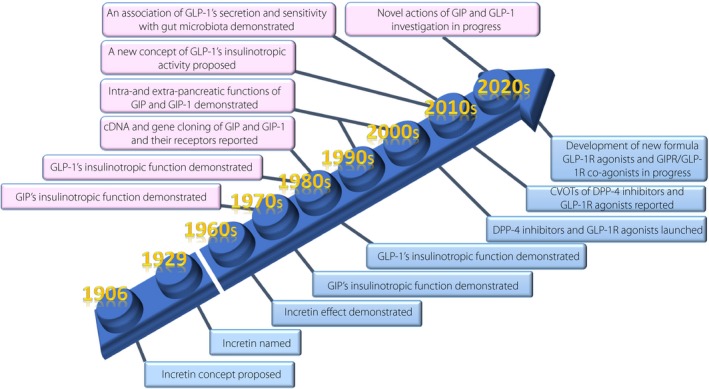
The journey to understanding incretin systems in human and experimental models. Events in humans are labeled by blue; those in experimental models by pink. cDNA, complementary deoxyribonucleic acid; CVOTs, cardiovascular outcome trials; DPP‐4, dipeptidyl peptidase‐4; GIP, glucose‐dependent insulinotropic polypeptide; GIPR, glucose‐dependent insulinotropic polypeptide receptor; GLP‐1, glucagon‐like peptide‐1; GLP‐1R, GLP‐1 receptor.

Initially, it was recognized that both GIP and GLP‐1 are secreted into systemic circulation from the gut, and directly activate their receptors expressed on pancreatic β‐cells to stimulate insulin secretion as intestinal hormones^5^. However, recent studies in experimental models showed that GLP‐1 exerts its insulinotropic activity through two distinct mechanisms: (i) gut‐derived GLP‐1 activates receptors expressed in nodose ganglions, thereby potentiating glucose‐dependent insulin secretion through the vagus nerve; and (ii) pancreatic α‐cells secrete GLP‐1 that activates receptors expressed in β‐cells in a paracrine manner^5^. The classical experiments to show the insulinotropic activity of GLP‐1 were carried out by intravenous infusion of gut extracts or recombinant GLP‐1 at pharmacological levels; they did not address the mechanism by which gut‐derived GLP‐1 reaches the pancreatic β‐cells through systemic circulation. Although half the maximal effective concentration of the GLP‐1 receptor for cyclic adenosine monophosphate production is 10–100 pmol/L, the physiological levels of biologically intact GLP‐1 in plasma are usually <10 pmol/L, even after meal ingestion with DPP‐4 inhibitors^5^. This novel concept might explain why DPP‐4 inhibitors exert their substantial glucose‐lowering effects: DPP‐4 inhibitors might increase biologically intact GLP‐1 locally in the gut, as well as in the pancreatic islets to strongly activate insulin secretion.

Recently, a link between GLP‐1 and the gut microbiome in diabetes has been receiving attention. GLP‐1‐secreting L cells are distributed throughout the intestine^6^. Some gut microbiota produce short‐chain fatty acids, thereby increasing the number of L cells in the gut and increasing GLP‐1 secretion^7^, ^8^. Gut microbiota dysbiosis, in contrast, results in downregulation of GLP‐1 receptors in nodose ganglions and decreases insulin secretion^9^. As gut microbiota are known to be highly influenced by daily dietary habits^10^, ^11^, they might well affect the efficacy of DPP‐4 inhibitors. Recently, it was shown that excess saturated fat intake impairs the glucose‐lowering effects of DPP‐4 inhibitors, possibly by enhancing GIP secretion^12^. It would be interesting to investigate the effects of daily dietary habits on the gut microbiome and GLP‐1 secretion and sensitivity to improve the efficacy of DPP‐4 inhibitors.

Studies of the extrapancreatic functions of GLP‐1 showed potential benefits of DPP‐4 inhibitors, as well as GLP‐1 receptor agonists in the management of diabetes and its complications independently of glycemic control^13^. Indeed, cardiovascular outcome trials showed that some GLP‐1 receptor agonists exert cardiovascular and renal benefits, whereas such benefits could not be confirmed for DPP‐4 inhibitors^14^. These cardiovascular outcome trials often recruited type 2 diabetes patients with a history of cardiovascular diseases or with multiple cardiovascular risks to complete trials in limited time periods; the sample sizes of these trials were designed to assess cardiovascular safety, but not other adverse events with relatively low incidence rates (e.g., acute pancreatitis)^14^. Thus, potential cardiovascular benefits of DPP‐4 inhibitors must await further investigations with appropriate surrogate markers, such as intima‐media thickening^15^; novel DPP‐4 inhibitors‐associated adverse events, such as bullous pemphigoid, must be carefully examined using large clinical databases^16^.

Accumulating clinical data on incretin‐based DPP‐4 inhibitors and GLP‐1 receptor agonists in the past decade have clearly confirmed their safety and efficacy as antidiabetes drugs. However, novel findings from basic research on GLP‐1, as well as GIP, are still changing our views on how incretin‐based drugs exert their glucose‐lowering effects, and how such effects can be maximized by lifestyle modifications. In contrast, newer incretin‐based drugs, including unimolecular peptide‐based dual agonists against GLP‐1 and GIP receptors, are providing clues as to how pharmacological levels of GIP affect appetite loss and bodyweight reduction^17^. Although it has been >100 years since the discovery of the incretin concept by Moore *et al*.^4^, the journey to understand the incretin system and its role in health and disease continues.

## Disclosure

D Yabe received consulting or speaker fees from MSD K.K., Novo Nordisk Pharma Ltd. and Taisho Toyama Pharmaceutical Co. Ltd. D Yabe also received clinically commissioned/joint research grants from Taisho Toyama Pharmaceutical Co. Ltd., Ono Pharmaceutical Co. Ltd., Novo Nordisk Pharma Ltd., Arklay Co. Ltd. and Terumo Co. Ltd. H Kuwata received clinically commissioned/joint research grants from Taisho Toyama Pharmaceutical Co. Ltd., Ono Pharmaceutical Co. Ltd., Novo Nordisk Pharma Ltd., Arklay Co. Ltd. and Terumo Co. Ltd. Y Seino received consulting or speaker fees from Eli Lilly Japan K.K., Sanofi K.K., Novo Nordisk Pharma Inc., Glaxo‐Smith‐Kline, Taisho Pharmaceutical Co., Ltd., Taisho Toyama Pharmaceutical Co., Ltd., Astellas Pharma Inc., BD, Nippon Boehringer Ingelheim Co., Ltd., Johnson & Johnson and Takeda Pharmaceutical Company Limited. Y Seino also received clinically commissioned/joint research grants from Nippon Boehringer Ingelheim Co., Ltd., Eli Lilly and Company, Taisho Toyama Pharmaceutical Co. Ltd., MSD K.K., Ono Pharmaceutical Co. Ltd., Novo Nordisk Pharma Ltd., and Arklay Co. Ltd.
